# GIS automated multicriteria analysis (GAMA) method for susceptibility modelling

**DOI:** 10.1016/j.mex.2019.10.031

**Published:** 2019-11-02

**Authors:** Fran Domazetović, Ante Šiljeg, Nina Lončar, Ivan Marić

**Affiliations:** Department of Geography, Center for Karst and Coastal Researches, Geospatial Analysis Laboratory, University of Zadar, Trg kneza Višeslava 9, 23000, Zadar, Croatia

**Keywords:** GAMA (GIS automated multicriteria analysis), ModelBuilder, ArcGIS, Automated GIS-MCDA, Gully erosion, GAMA

## Abstract

Developed GIS automated multicriteria analysis (GAMA) method presented in this article allows automation and simplification of multicriteria GIS decision analysis (GIS-MCDA) susceptibility modelling. Traditional GIS-MCDA susceptibility modelling still represents time and labor demanding process, whose success is highly influenced by the user's experience and knowledge. In order to make overall GIS-MCDA susceptibility modelling process more straightforward and practical, GAMA method is designed as easy to use three step process, which allows automation of following GIS-MCDA steps: (1) *standardization of criteria*, (2) *criteria grouping and weight assignment* and (3) *susceptibility model aggregation*. GAMA method is developed within ArcGIS 10.4 ModelBuilder application, as a toolset that can be easily shared and incorporated within default ESRI’s ArcGIS toolbox. GAMA was successfully applied for gully erosion susceptibility modelling on example of Pag island, Croatia, whose results are published in separate article. Since GAMA method is applicable to various GIS-MCDA susceptibility modelling purposes we are encouraging its future use and therefore open-source GAMA method toolset can be acquired for research purposes (htps://gal.unizd.hr).

• GAMA method simplifies overall GIS-MCDA susceptibility modelling process.
• GAMA allows automated standardization, grouping, weight coefficient assignment and aggregation of GIS-MCDA’s criteria.
• GAMA method has broad application in various different GIS-MCDA susceptibility modelling purposes.

## Graphical abstract

**Figure:**
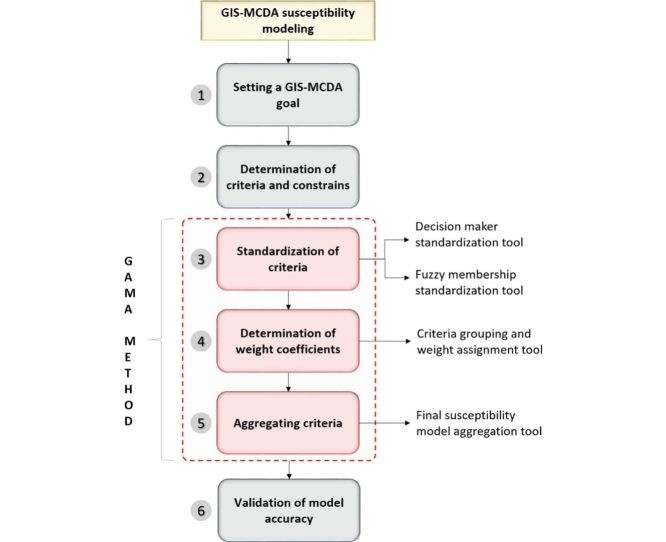

**Specification Table**

**Table:** 

Subject Area:	Environmental Science
More specific subject area:	Soil erosion research
Method name:	GAMA (GIS Automated Multicriteria Analysis)
Name and reference of original method:	GIS-Based Multicriteria Decision Analysis
Resource availability:	Developed GAMA toolset is available freely through the official web page of Geospatial Analysis Laboratory (gal.unizd.hr).

## Method details

Multicriteria GIS Decision Analysis (GIS-MCDA) is one of the most commonly used methods for susceptibility modelling that has application in various scientific fields [1]. Therefore, it is not surprising that in recent years there has been a significant increase in the number of published researches, that have used GIS-MCDA for different susceptibility modelling purposes [[2], [3], [4], [5]]. However, traditional GIS-MCDA susceptibility modelling still represents time and labor demanding process, whose success is highly influenced by the user's decisions, experience and knowledge. In order to make overall GIS-MCDA susceptibility modelling process more straightforward and practical, GAMA method is designed as easy to use three step process that reduces and simplifies required processing. In general, GIS-MCDA consists of following six substeps: setting a goal (1), determination of criteria and constraints (2), standardization of criteria (3), determination of weight coefficients (4), aggregating criteria (5) and validation of model accuracy (6) [6]. Developed GAMA method allows automation of steps 3, 4 and 5, which are explained in detail below. First two steps (1 and 2) of GIS-MCDA process are depending on the user’s preferences and available data, while last step, validation of model accuracy can be performed in many ways and depends highly on available reference data. Therefore, these GIS-MCDA steps are excluded from developed GAMA method and had to be performed separately by the user prior (steps 1 and 2) and after (step 6) to the application of GAMA method.

GAMA method is developed within ArcGIS 10.4 ModelBuilder application [7], as a toolset that can be easily shared and incorporated within default ESRI’s ArcGIS toolbox. While few other tools for semi-automation of GIS-MCDA process currently exist within commercial (e.g. extAHP 2.0, gdt_SAR or gdTools for ArcGIS) or open-source (e.g. VectorMCDA for QGIS) GIS software, these tools are either concentrated on one specific step of GIS-MCDA workflow, or they have some important shortcomings, e.g. limitation of maximal number of criteria. As an example, extAHP 2.0 tool performs calculation of criteria weight coefficients and determination of criteria hierarchy, but it is limited to processing of maximum 15 criteria and lack tools for integrated criteria standardization [8]. VectorMCDA implements several MCDA algorithms for susceptibility modelling within QGIS software, but it is developed for vector data, and because of that it cannot be used in raster susceptibility modelling [9]. These shortcoming are resolved through the development of GAMA method. Within this research GAMA method was used for modelling of gully erosion susceptibility, based on 10 erosion predisposing criteria and one constraining (Boolean) factor. Gully erosion susceptibility modelling was performed on example of Pag island, Croatia, and research results are published in separate article [10], while this article concentrates entirely to the explanation of the developed GAMA method. Since GAMA method is applicable to various GIS-MCDA susceptibility modelling purposes we are encouraging its future use and therefore open-source GAMA method toolset can be acquired for research purposes through the official web page of Geospatial Analysis Laboratory (htps://gal.unizd.hr).

### Structure of developed GAMA method

**Fig. 1:**
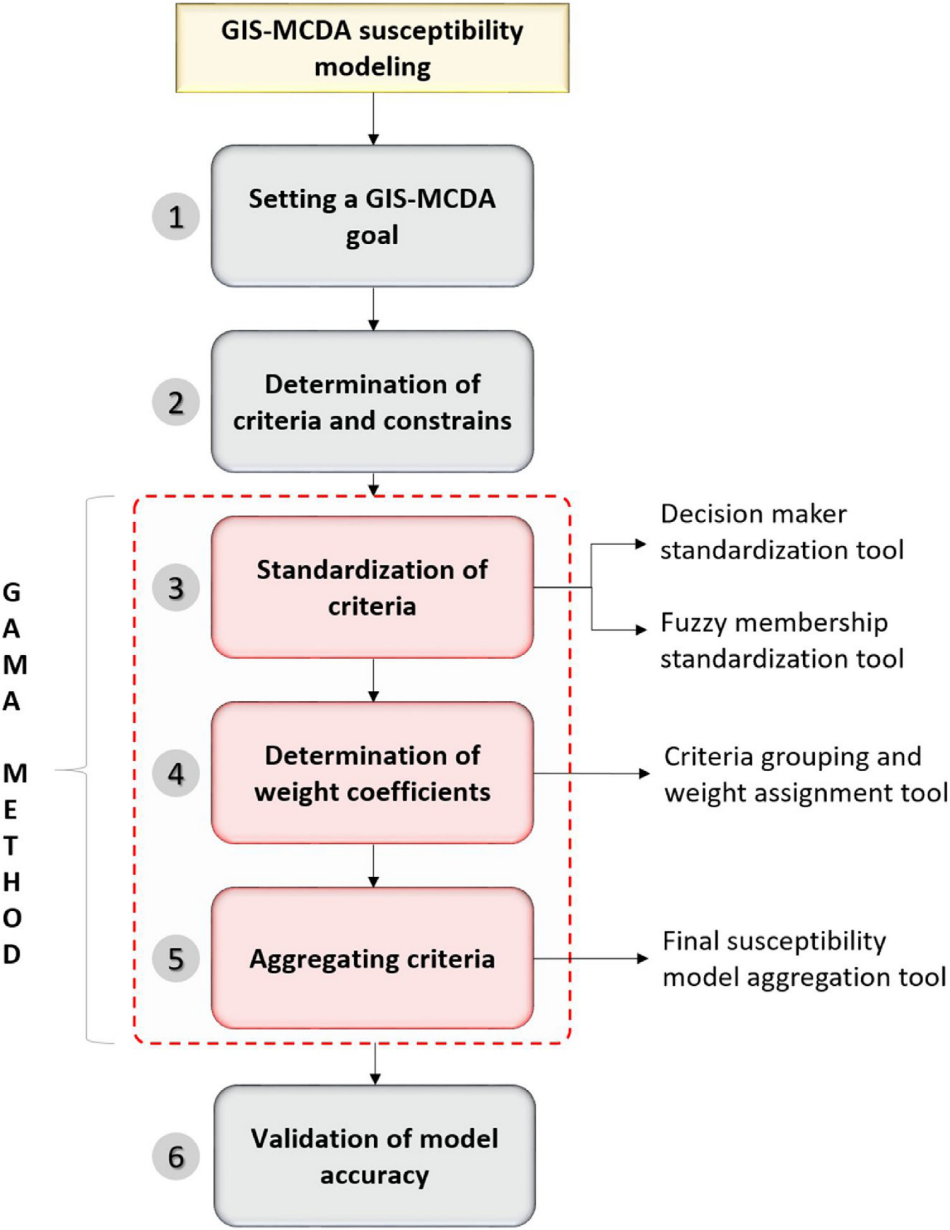
Three steps (3–5) of GIS-MCDA workflow automated by GAMA method.

GAMA method can be divided into three separate steps, that can be applied either as a series of automated steps integrated within whole GIS-MCDA process, or as separate tools that fulfill just one specific function. In other words, user can either use whole GAMA toolset to automate steps 3–5 of GIS-MCDA process, or use just one specific tool to perform required task (e.g. only standardization of criteria). Structure of GAMA method integrated within overall GIS-MCDA process can be seen in Fig. 1, while detailed explanation of its every step is explained below.

### Step 1. Criteria standardization

**Fig. 2:**
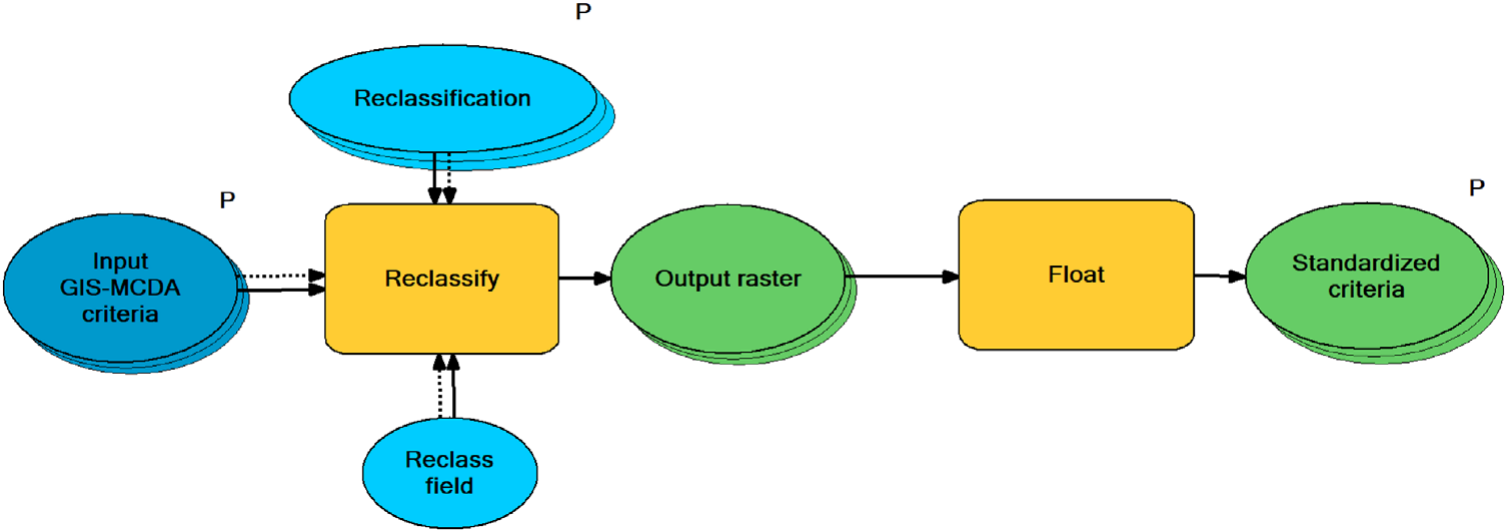
Structure of *Decision maker standardization (DMS)* tool.

Standardization of criteria is first step within developed GAMA method, through which all input raster-based criteria chosen by the user are being standardized to the same scale (e.g. 1–5; 0–1). Within GAMA toolbox standardization can be performed by two different approaches. First approach is *Decision maker standardization (DMS) tool* (Fig. 2) through which different classes of one criteria are standardized to the scale from 1 to 5, on the basis of decision maker’s experience-based suitability evaluation. In this approach lower values are representing less suitable areas (1 – very low suitability; 2 – low suitability), value 3 represents medium suitability, while higher grades (4- high suitability, 5 – very high suitability) represent more suitable areas for certain process. Through the DMS tool user can automatize standardization of unlimited number of criteria, while reclassification values had to be set for every criteria separately. Accuracy of this standardization approach is depending on the quality of the decision maker’s evaluation that reflects his expertise and knowledge.

**Fig. 3:**
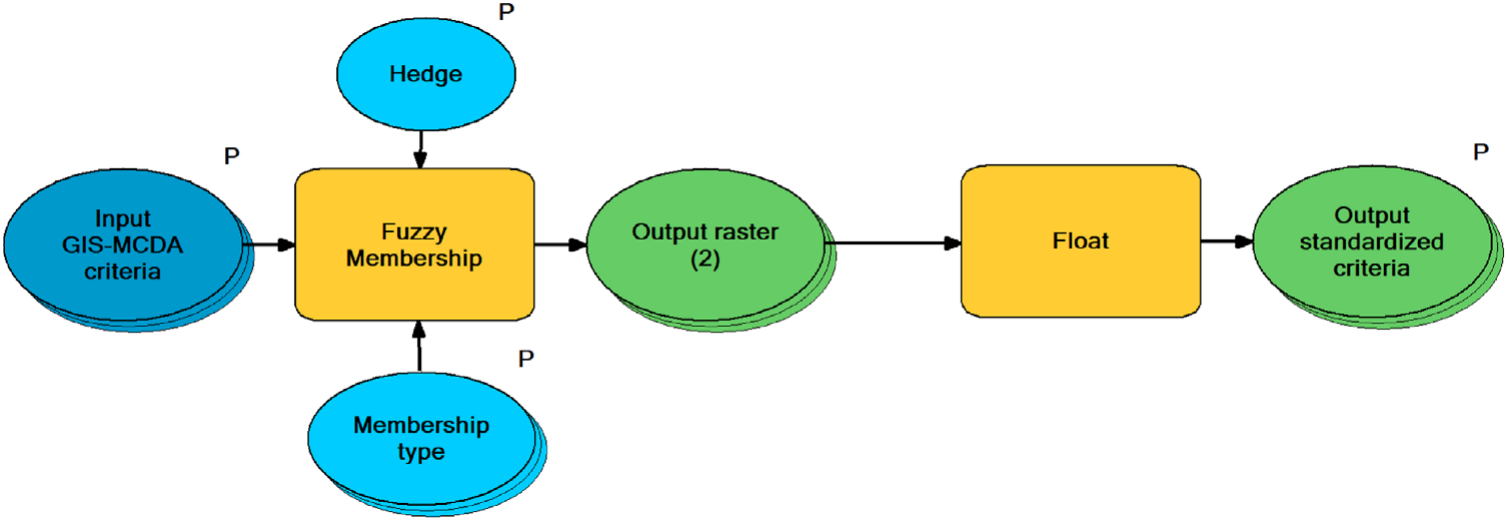
Structure of *Fuzzy membership standardization (FMS)* tool.

**Fig. 4:**
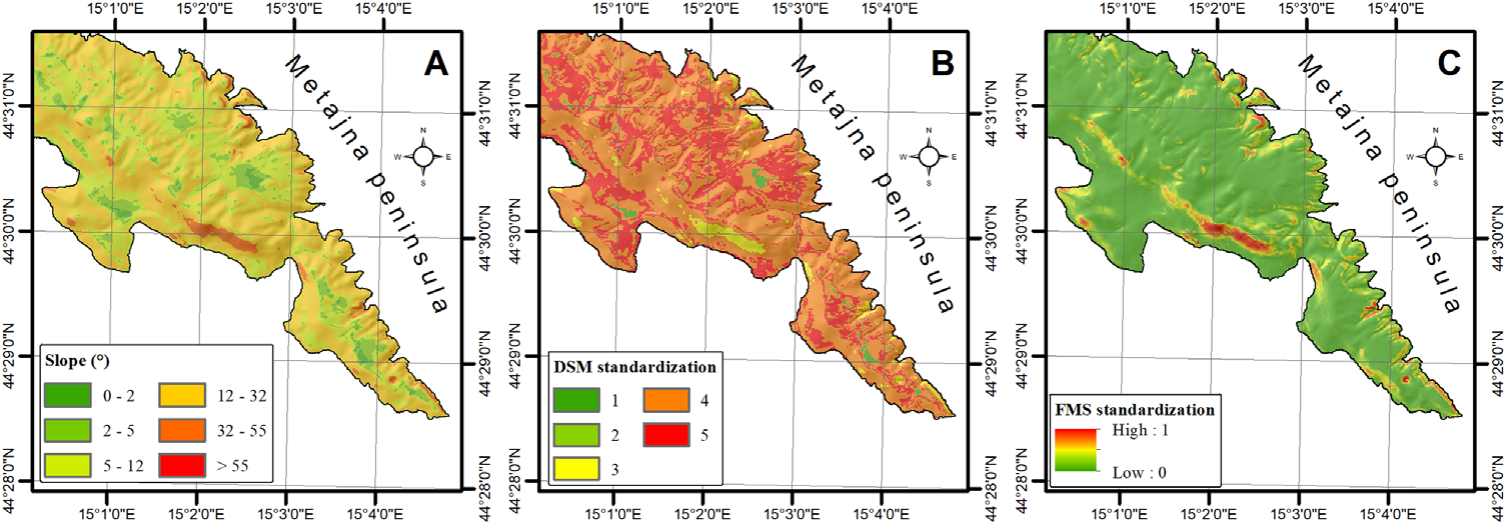
Initial slope criteria (A); slope criteria standardized by DSM tool (B); slope criteria standardized by FMS tool (C) for study area of Metajna peninsula, Pag Island, Croatia.

Second standardization approach that is available through the GAMA method is fuzzy membership standardization (FMS) tool (Fig. 3) which allows standardization of criteria to the scale from 0 to 1 based on the one of seven possible user-defined fuzzy membership types (*Gaussian, Small, Large, Near, MS Small, MS Large, Linear*). Fuzzy membership is common standardization method within GIS-MCDA application, which allows logic standardization of criteria, where suitable membership type can be selected by the user according to the type of criteria that is being standardized. FMS tool allows automated standardization of unlimited number of criteria, while user has to choose appropriate fuzzy membership type for every criteria. Selection of membership type is the most important part of FMS tool application, because it directly influences the standardization results. In Fig. 4 comparison between original criteria (Fig. 4A) and criteria standardized by DMS tool (Fig. 4B), or by the FMS tool (Fig. 4C) can be seen. Difference between results generated by DMS and FMS tools is evident, therefore, selection of optimal method should be based on criteria type and characteristics, as well as on user’s experience and preferences.

### Step 2. Criteria grouping and weight assignment

Second step within developed GAMA method includes grouping of standardized criteria and assignment of calculated criteria weight coefficients, through the application of *Criteria grouping and weight assignment (CGW) tool*. This tool allows user to distribute standardized criteria in two or more criteria groups and to assign unique weight coefficients to every criteria within created criteria groups. Many different weight coefficient calculation (CWC) methods exist (e.g. *simple multi-attribute rating technique* (SMART), *trade-off method*, *swing method*, *point allocation method*, *direct rating method*, *analytic hierarchy process* (AHP), *best worst method* (BWM), *full consistency method* (FUCOM), etc.) [11,12]. The process of developing a tool for CWC is complex and has been a separate subject of research in numerous scientific papers [[13], [14], [15]]. All existing CWC methods have certain advantages and disadvantages, depending on the research aims and purpose [11]. Generally, one of the most frequently used CWC method is analytical hierarchical process (AHP) [[16], [17], [18]], which was successfully applied within application of GAMA method for gully erosion susceptibility modelling [10]. For complex comparisons of different CWC methods, sensitivity analysis and model validation, we suggest using simpler tools (e.g. DAME [15]) or advanced software solutions (e.g. Definite 3.1 [19]; DEXi 5.02 [20], M-Macbeth [21], etc.). The developed GAMA method is not limited and is compatible with all above mentioned tools and advanced software solutions and therefore selection of optimal CWC method should be performed by the user prior to the application of GAMA method.

**Fig. 5:**
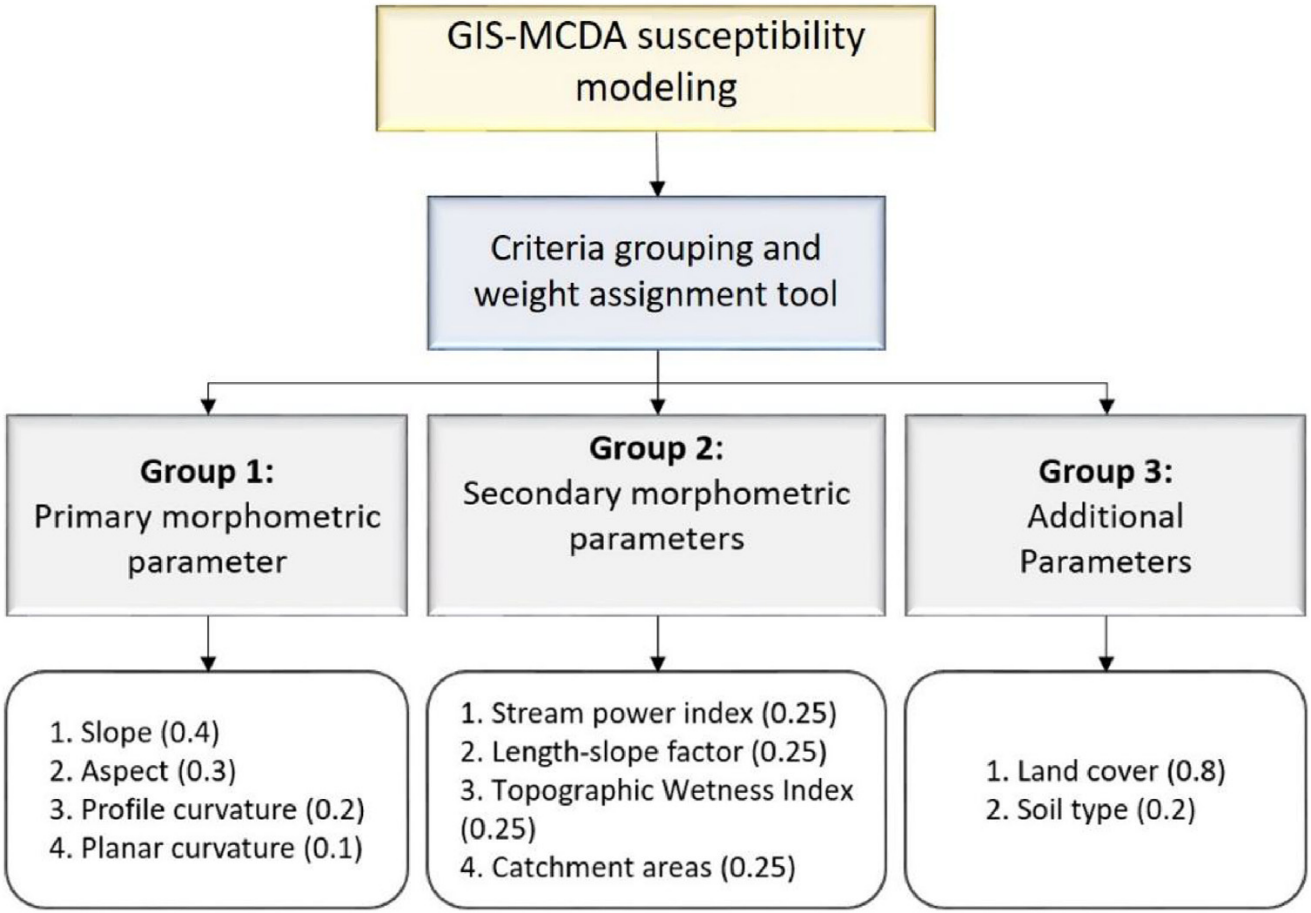
Example of 10 standardized criteria with assigned weight coefficients that are grouped into three main criteria groups by CGW tool.

Criteria grouping allows mutual comparison of all criteria within one group through assignment of unique weight coefficients to every chosen GIS-MCDA criteria. Criteria distributed within one group are hierarchically compared and ranked by their importance for susceptibility modelling expressed through assigned unique weight coefficients, where sum of all weight coefficients within one group equals to 1. As an example (Fig. 5)^1^, 10 erosion predisposing criteria that were used for gully erosion susceptibility modelling were grouped into three criteria groups: *primary morphometric parameters* (slope, aspect, profile and planar curvature), *secondary morphometric parameters* (topographic wetness index, stream power index, length-slope factor, catchment area) and *additional parameters* (soil type and vegetation cover), where different unique weight coefficients were assigned to every criteria within three groups.

**Fig. 6:**
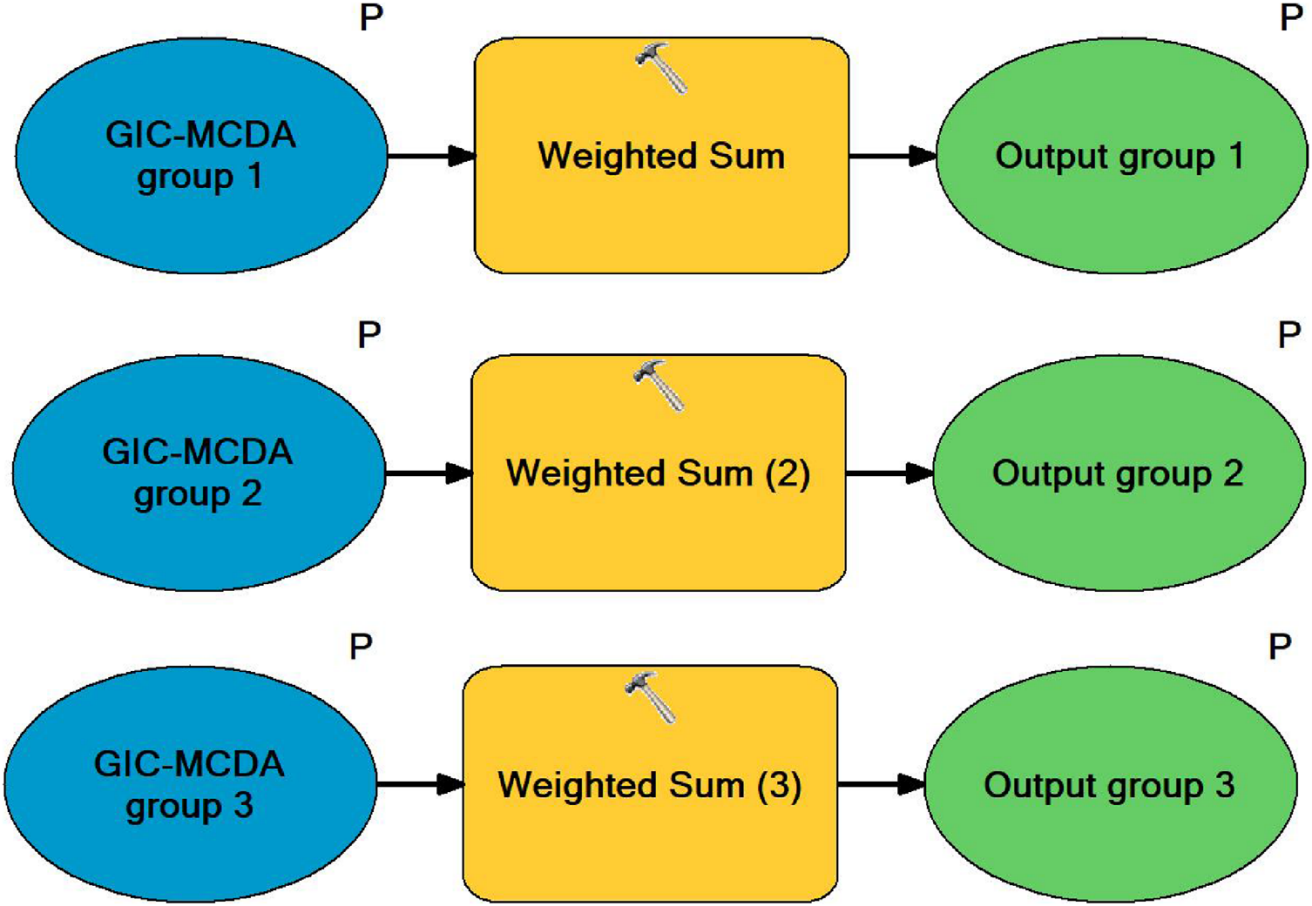
Structure of Criteria grouping and weight assignment (CGW) tool.

Since only three criteria groups were used for modelling of gully erosion susceptibility, created *CGW* tool can perform grouping of standardized criteria into three criteria groups (Fig. 6). But structure of CGW tool can be easily edited within ArcGIS’s ModelBuilder application, if higher number of criteria groups is required for susceptibility modelling. In order to add one additional criteria group, it is only necessary to open CGW tool editing window within ModelBuilder application and to copy one Weighted Sum procedure for each required additional criteria group. In that way CGW tool can be easily modified to allow distribution of GIS-MCDA criteria in four or more criteria groups.

### Step 3. Susceptibility model aggregation

**Fig. 7:**
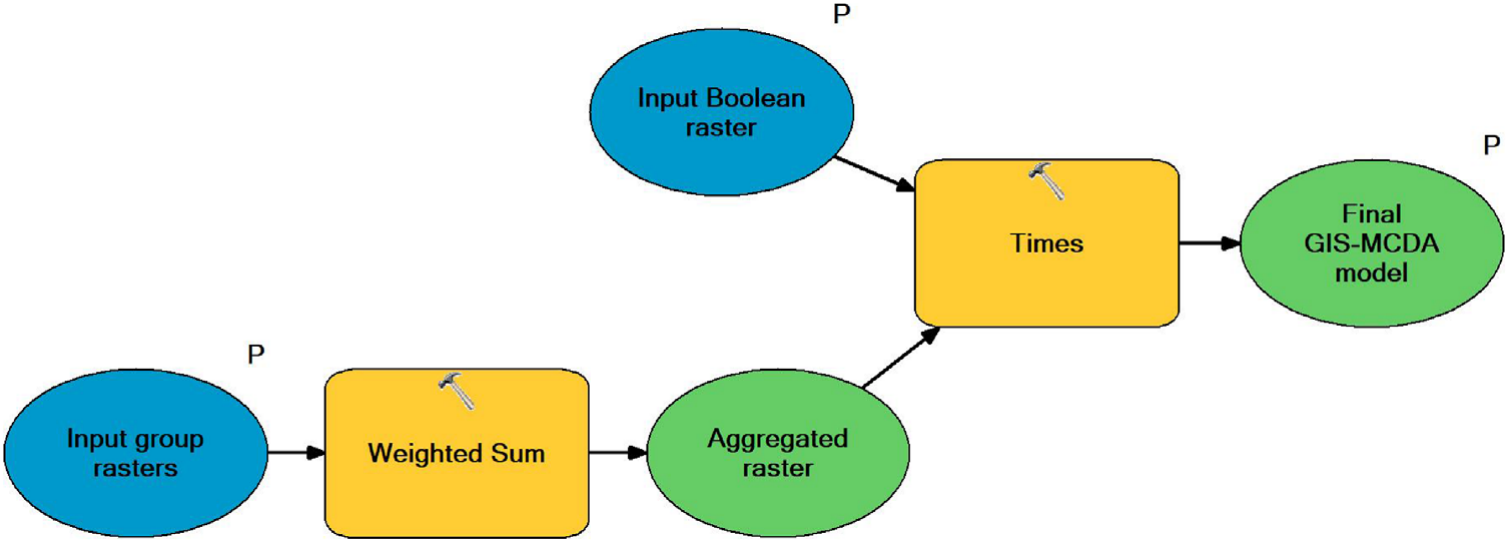
Structure of Susceptibility model aggregation (SMA) tool.

**Fig. 8:**
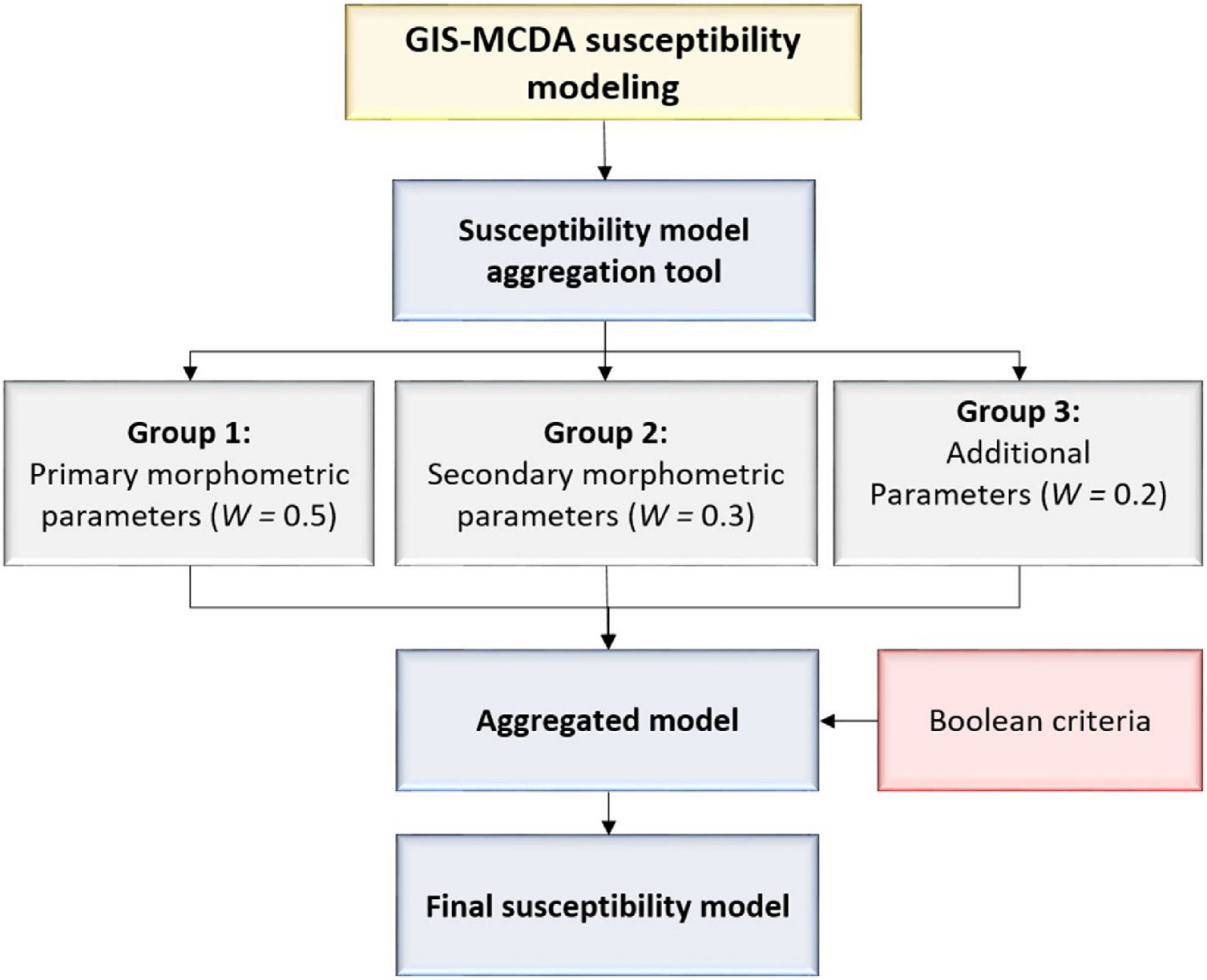
Aggregation of criteria groups and weight coefficients with constraining (Boolean) criteria to produce final susceptibility model.

Last step of GAMA method application includes aggregation of created criteria groups with Boolean criteria in order to produce final susceptibility model, through the application of S*usceptibility model aggregation* (SMA) *tool* (Fig. 7). Aggregation of constraining Boolean criteria with predisposing criteria groups allows removal of all unsuitable areas from final susceptibility model. For an example with this option all unsuitable areas for gully erosion occurrence (e.g. water bodies, urban areas) were removed from final created gully erosion susceptibility model of Pag island. Additionally, before the aggregation of criteria groups different weight coefficients can be assigned to each group, according to its importance for susceptibility modelling, where total sum of weights coefficients of all criteria groups has to be equal to 1. As an example (Fig. 8)^2^, three criteria groups used for gully erosion susceptibility could be hierarchically ranked according to their importance for erosion susceptibility, where *Criteria group 1* is the most important and therefore has highest weight coefficient (*W* = 0.5) assigned, while smaller weight coefficients are assigned to less important criteria groups: *Criteria group 2* (*W* = 0.3) and *Criteria group 3* (*W* = 0.2). Quality and accuracy of final susceptibility model created by SMA tool can be further validated through one of common validation methods (e.g. receiver operating characteristic (ROC) curves, success and prediction rate curves, sensitivity analysis, etc.).

## Conclusion

GAMA method represents simple, practical and enhanced approach for standardization, grouping, weight coefficient assignment and aggregation of GIS-MCDA’s criteria, which can process unlimited number of criteria. Due to the developed GAMA method criteria standardization is automated, where user has to choose standardization approach and define standardization parameters, after which the processing of all criteria is autonomous. This shortens overall processing time, in comparison to individual standardization of each chosen criteria. Furthermore, GAMA allows distribution of standardized criteria in unlimited number of criteria groups, while it is possible to assign unique weight coefficient to each criteria within created groups. This allows organization and hierarchical comparison of all GIS-MCDA criteria within the created criteria groups. Finally, GAMA allows aggregation of all created criteria groups and their corresponding weight coefficients with the constraining Boolean criteria. GAMA was successfully applied for gully erosion susceptibility modelling on the example of Pag island, Croatia. However, due to its adaptability and versatility GAMA can be applied in broad range of GIS-MCDA susceptibility modelling purposes (e.g. urban planning, hazard prediction, habitat suitability modelling, agricultural suitability, risk management, etc.).

## Declaration of Competing Interest

The authors declare that they have no known competing financial interests or personal relationships that could have appeared to influence the work reported in this paper.

## Footnotes

1 W*eight coefficients used in this example are explanatory and they do not correspond to weight coefficients used in GIS-MCDA modelling of gully erosion susceptibility.*

2 W*eight coefficients used in this example are explanatory and they do not correspond to weight coefficients used in GIS-MCDA modelling of gully erosion susceptibility.*
